# Three-Dimensional Meso-Structure-Based Model for Evaluating the Complex Permittivity of Asphalt Concrete

**DOI:** 10.3390/ma17081900

**Published:** 2024-04-19

**Authors:** Zhenwen Xie, Xingzao Chen, Jing Wang, Jiaqi Chen

**Affiliations:** 1 Hunan Construction Investment Group Co., Ltd., Changsha 410004, China; zwxie_hcig@163.com; 2 Department of Civil Engineering, Central South University, Changsha 410075, China; chenxingzao@csu.edu.cn (X.C.); wangjing21@csu.edu.cn (J.W.)

**Keywords:** asphalt mixture, complex permittivity, dielectric model, meso-scale model, finite element

## Abstract

Microwave heating is an emerging alternative pretreatment method for road maintenance in cold seasons. The thermal behavior of asphalt pavement under microwave heating is mainly determined by the complex permittivity of the asphalt mixture. In this study, an innovative approach for calculating the complex permittivity of an asphalt mixture based on a three-dimensional meso-scale heterogeneous structure was proposed. A series of experiments was conducted to verify the accuracy of this approach. The effect of porosity, void size, moisture content and aggregate gradation on the complex permittivity for an asphalt mixture were computationally analyzed based on the validated approach. Moreover, the applicability of commonly used classical dielectric models was analyzed. The classical Lichtenecker–Rother (LR) dielectric model was modified on the basis of simulation data for various conditions. The results showed that the real part of the complex permittivity decreased with the increase in porosity. Some sudden change in the imaginary part of the complex permittivity was observed within the frequency range from 2.6 GHz to 3.9 GHz. A larger air void size would lead to a larger frequency at which sudden change occurs. The real part and imaginary part of the complex permittivity tend to be smaller when more coarse aggregates are replaced with fine aggregates. Both the real part and the imaginary part of the complex permittivity increase with higher moisture content due to the stronger dielectric property of water. Each 1% increase in moisture content would lead to about a 3~4% increase in the real part of the complex permittivity. The determination coefficients R^2^ for the real part and the imaginary part of the complex permittivity fitted by the modified Brown model were the maximum values, which were 0.922 and 0.980, respectively. The method presented in this study is useful for transportation agencies to optimize microwave heating during winter maintenance.

## 1. Introduction

The low temperature of asphalt pavement in winter would lead to a poor fluidity of asphalt binder, which is adverse to asphalt pavement’s repair and maintenance. Microwave heating is an emerging alternative pretreatment method for road maintenance in cold seasons due to its non-polluting, deep penetrating and rapid heating characteristics [1,2,3]. By converting the microwave energy into thermal energy [4], the microwave heats asphalt pavement with different depths [5], enabling the asphalt mixture to maintain fluidity during the repairment in a cold season. Since materials have a significant effect on the efficiency of microwave heating [6,7], investigating the dielectric behavior of asphalt concrete is important for optimizing microwave heating.

The dielectric behavior of asphalt concrete depends on its complex permittivity. At present, there are various approaches to measure the permittivity of asphalt concrete. The vector network analyzer, the dielectric constant test platform for relative electric permittivity and Percometer measurement [8,9,10] are mostly used in laboratories to measure the permittivity of asphalt concrete. Yu et al. developed the relative humidity–frequency equivalence principle for the dielectric properties of asphalt mixtures based on experiments [11]. Frid et al. obtained the relative electric permittivity of asphalt concrete with different moisture levels through experiments [12]. Yu et al. studied the effect of temperature on the dielectric properties of asphalt concrete, and analyzed the relative electric permittivity and dielectric loss of limestone, asphalt binder and asphalt concrete at different temperatures [13]. Jaselskis et al. described the dielectric properties of asphalt pavement at various frequencies and temperatures [14]. Liu et al. investigated the effect of microwave-absorbing materials such as magnetite powder and graphite powder on the overall complex permittivity through experiments [15,16]. Trigos et al. tested the permittivity, loss factor and loss tangent of aggregates with 26 different sources to analyze their sensitivity to microwaves [17]. Zhai et al. investigated the influence of various components in mineral aggregates with different particle sizes on the dielectric response of asphalt concrete using multi-scale experimental data and micromechanical methods [18]. Although experimental measurement is a straightforward method for evaluating the dielectric behavior of asphalt mixtures, it is usually time-consuming considering the repeatability of measurements and preparation of test specimens. From this view, models that predict complex permittivity based on material composition may have potential advantages over experimental methods.

The commonly used classic dielectric models mainly include the Lichtenecker–Rother (LR) model and the Rayleigh model [8], while the LR model can be divided into three types: the general linear model (Brown model), the root-mean-square model (CRIM model) and the cube root model (Looyenga model). Based on these classic models, some improved dielectric models, which are more suitable for asphalt concrete, have also been presented. Yu et al. developed a dielectric model for asphalt concrete based on the measured relative electric permittivity data under different frequencies and relative humidities [11]. Zhang et al. established new dielectric models based on experiments, including an improved Rayleigh model and LI model [19]. Piuzzi et al. analyzed the applicability of the α model, the Ansoult model and the ToPP model in evaluating the impact of moisture content on permittivity [20]. Cao developed a heterogeneous numerical model to simulate AC pavement with internal moisture at various saturation levels, which aimed to study the effect of moisture content on the dielectric properties of asphalt pavement [21]. Zhong et al. studied the dielectric properties of asphalt mortar and developed a dielectric model for asphalt mortar considering the impact of temperature [22]. Zhong et al. developed a dielectric model suitable for four types of asphalt concrete based on classic models and linear regression analysis [23]. These dielectric models could provide the approximate relationships between permittivity and some critical factors. However, it is usually difficult for these models to consider the effect of the complex internal structure of asphalt concrete, such as the spatial distribution of aggregates and air voids.

The meso-structure of asphalt concrete has a significant influence on the overall properties. In recent years, extensive studies have been conducted to investigate the effect of meso-scale structure on the mechanical and thermal properties of asphalt concrete [24,25,26,27,28]. Although some two-dimensional (2-D) meso-structure models for evaluating the dielectric properties of asphalt concrete were also reported in the existing literature [29], the heterogeneous internal structure of asphalt concrete was overly simplified, which limited the applicability of these models. Therefore, three-dimensional (3-D) meso-structure models for evaluating the complex permittivity of asphalt concrete considering the heterogeneous internal structures of asphalt concrete are needed.

## 2. Objectives

The objective of this study is to develop a 3-D meso-structure-based model for evaluating the complex permittivity of asphalt concrete. A multi-scale hierarchical modelling strategy was used to construct the virtual heterogeneous structure of asphalt concrete considering random distributions of components. The complex permittivity of the virtual specimens was numerically calculated in COMSOL. Moreover, the accuracy of the meso-structure model was validated with experiments conducted in this study.

## 3. Virtual Test Modelling for Complex Permittivity

### 3.1. Meso-Structure Modelling for Asphalt Concrete

In this section, a method for evaluating the complex permittivity based on the meso-structure of asphalt concrete was proposed. In this method, the asphalt concrete was regarded as a heterogeneous composite consisting of coarse aggregates, fine aggregate matrix (FAM) and air voids. The coarse aggregates referred to aggregates with a particle size greater than 2.36 mm. The FAM was considered to be a continuous homogeneous mixture of asphalt binder, mineral fillers and fine aggregates with a particle size smaller than 2.36 mm. The generation of virtual asphalt mixture specimens could be divided into the following three steps.

(1) Generation of 3-D aggregates

**Figure 1:**
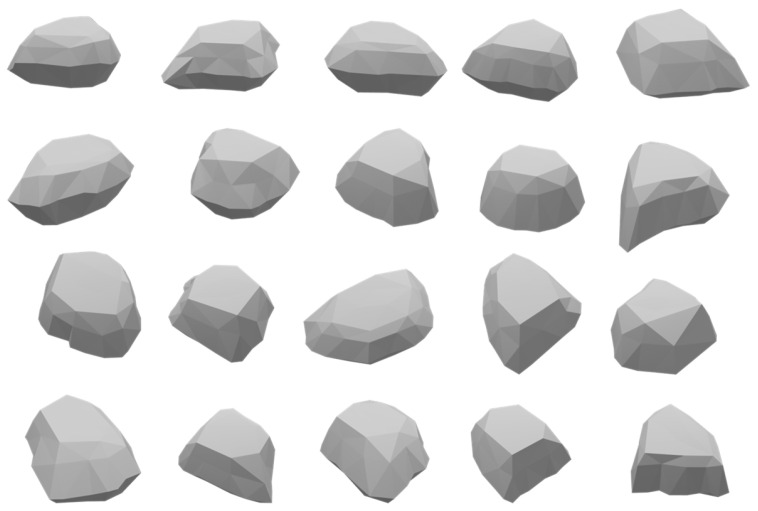
The schematic diagram of some 3-D geometric aggregate shapes.

An image-assisted approach was used to generate the geometries of 3-D aggregates. The Aggregate Image Analysis System (AIMS) was used to capture the 2-D projections of aggregates. To generate a 3-D aggregate with a 2-D projection, a series of points was generated inside the boundary of the 2-D projection; then, random coordinates along the normal direction of the projection were assigned to these points. These points with 3-D spatial coordinates were used to represent a 3-D aggregate. More detailed information on the above generation algorithm can be found in the authors’ previous study [30]. These 3-D digital aggregates were stored as a series of Standard Template Library (STL) files for future use. Figure 1 shows examples of generated 3-D aggregates.

(2) Generation of 3-D asphalt concrete specimen

**Figure 2:**
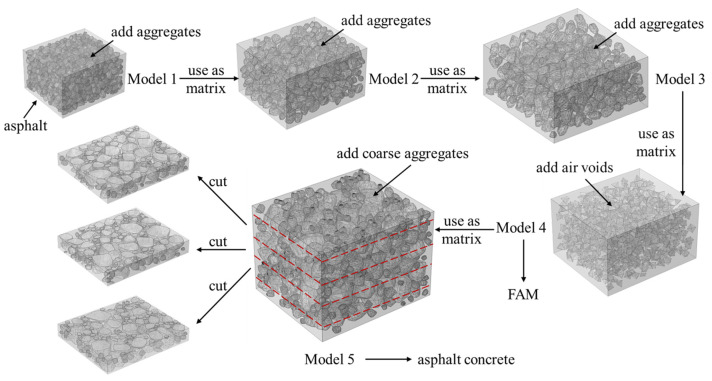
The schematic diagram for hierarchical modelling of asphalt concrete.

Since the aggregates sizes in the asphalt concrete range a lot, it is impossible to include all aggregates and air voids in one single model. In this section, a hierarchical modelling strategy, shown in Figure 2, was used to generate the 3-D asphalt concrete specimen.

As shown in Figure 2, aggregates smaller than 0.15 mm were added into asphalt binder to generate a mixture marked as Model 1. Then, this mixture (Model 1) was mixed with larger aggregates (sizes between 0.15 mm and 1.18 mm) to form a mixture marked as Model 2. Similarly, Model 2 was used as the matrix of Model 3, which contained larger aggregates (sizes between 1.18 mm and 2.36 mm). After that, air voids were added into the matrix represented by Model 3 to form the mixture of the FAM. The FAM is also marked as Model 4. In Model 4, the volume of the air void was determined based on the volume fractions of each component, including an asphalt content of 4.3% by weight, air void content of 6% by volume and fine aggregates. In addition, the air void was placed in Model 4 with the geometric shape of a quadrangular pyramid. By changing the random number, a virtual specimen with a random distribution of the air void was obtained. Finally, coarse aggregates larger than 2.36 mm were added into the FAM (Model 4) to generate the asphalt concrete specimen, namely, Model 5. To avoid non-uniformity at the boundaries of Model 5, three pieces of the specimen with a size of 70 mm × 60 mm × 10 mm were cut from the central part of Model 5. These three specimens were imported into COMSOL for further numerical analysis. During the above process, the asphalt content, air void and aggregate gradations were kept the same as those used in the experiment. The detailed material compositions and model dimensions for each model are summarized in Table 1.

(3) The calculation of scattering parameter (S-parameter)

**Figure 3:**
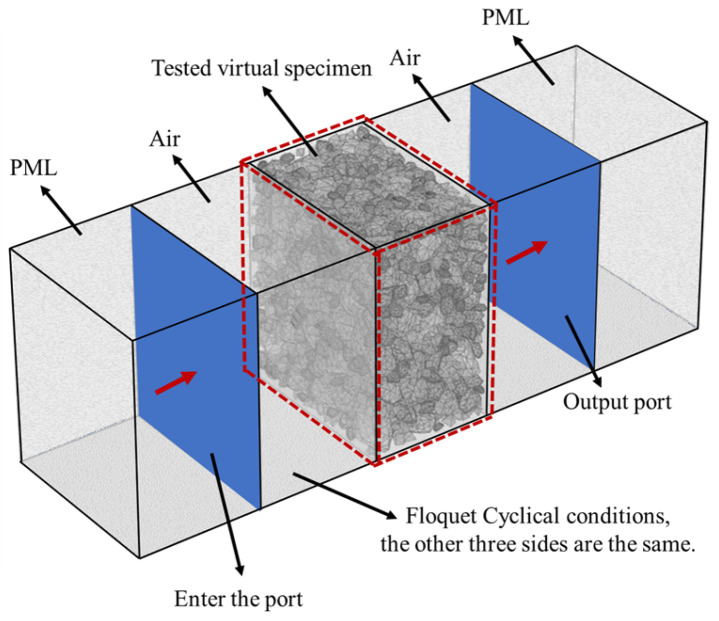
The electromagnetic wave model for 3-D meso-scale structure.

In this section, five cuboids with the same dimensions were produced to develop the electromagnetic wave model through the commercial software COMSOL Multiphysics (V6.0 FNL), as shown in Figure 3. The dimension of these models, shown in Figure 3, were consistent with the model dimensions shown in Table 1.

Based on the electromagnetic wave model, the S-parameter of each virtual 3-D specimen in this study was obtained. Then, the Nicolson–Ross–Weir (NRW) method was adopted to calculate the complex permittivity of each specimen. As shown in Figure 3, a Perfect Matched Layer (PML) was used in the leftmost and rightmost layer of the electromagnetic wave model, which aimed to absorb electromagnetic waves beyond the input and output ports. The two cuboids adjacent to the PML were considered to be filled with air. The interfaces between the air layer and the PML were considered to be the input and output ports for electromagnetic waves. The periodic ports were applied for more efficient calculation and the electromagnetic waves were vertically incident. The 3-D virtual specimen was located between the air layers. Moreover, Floquet Cyclical conditions were set on the normal plane of the electromagnetic wave transmission direction. The tetrahedral mesh was adopted in this model and the dimension of the tetrahedral mesh was determined by the frequency of electromagnetic waves.

### 3.2. The Theory for Calculating Complex Permittivity with S-Parameter

The S-parameters measured by the electromagnetic wave ports are not consistent with the S-parameters measured at both ends of the tested material. Based on the lossless transmission line theory, the modulus of reflection coefficients at both ends of the electromagnetic wave ports is equal to that of the tested material, but phase difference could be found. Therefore, the simulated S-parameter needs to be transformed. The complex permittivity could be calculated with the S-parameter on the basis of relevant electromagnetic theory [31] as follows:

(1) $$S_{11}=S_{11m}e^{\frac{4\pi jlf}{c}}$$

(2) $$S_{21}=S_{21m}e^{\frac{4\pi jlf}{c}}$$

where *S*_11_—Input reflection coefficient;

*S*_21_—Output reflection coefficient;
*S*_11m_—Input reflection coefficient on the electromagnetic wave port;
*S*_21m_—Output reflection coefficient on the electromagnetic wave port;
c—The velocity of light, m/s;
*f*—The frequency, GHz;
*l*—The length of air shed from the tested material to the port, m.

(3) $$A_{1}=S_{21}+S_{11}$$

(4) $$A_{2}=S_{21}-S_{11}$$

(5) $$X=\frac{\left(S_{11}^{2}-S_{21}^{2}+1\right)}{2S_{11}}$$

(6) $$I=X\pm \sqrt{X^{2}-1}$$

(7) $$T=\frac{A_{1}-I}{1-A_{1}I}$$

(8) $$Z=\frac{1+I}{1-I}$$

(9) $$\ln\left(T\right)=\ln\left(\left|T\right|\right)+j\left(\theta \pm 2m\pi \right),\;\;m=0,\;\pm 1,\;\pm 2,\;\cdots$$

(10) $$\varepsilon_{r}=n/Z$$

(11) $$n=\frac{\ln(T)}{jk_{0}l}$$

(12) $$\mu_{r}=n\times Z$$

(13) $$\varepsilon_{r}=\varepsilon^{\prime }-j\varepsilon^{\prime \prime }$$

where *I*—The reflection coefficient;

*T*—The propagation coefficient;
*θ*—The phase of T;
*n*—The refractive index;
*k*_0_—The wave vector in vacuum;
*ε*_r_—The complex permittivity of the specimen;
*ε*′—The real part of complex permittivity;
*ε*″—The imaginary part of complex permittivity;
*μ_r_*—The relative magnetic permeability.

The half-wave resonance and multi-valued problem may occur during the calculation process due to the different thicknesses of asphalt concrete. The multi-valued problem could be solved by choosing different values of *m* in Equation (9) reasonably (*m* = [*l*/*λ*_0_], [*x*] represents the maximum integer which is no more than *x*). The half-wave resonance problem could be solved by multiplying the complex permittivity and the complex magnetic conductivity. The transformed S-parameter could be used to calculate the complex permittivity of asphalt concrete with Equations (1)–(13).

## 4. Experiment

### 4.1. Materials and Specimens in the Experiment

**Figure 4:**
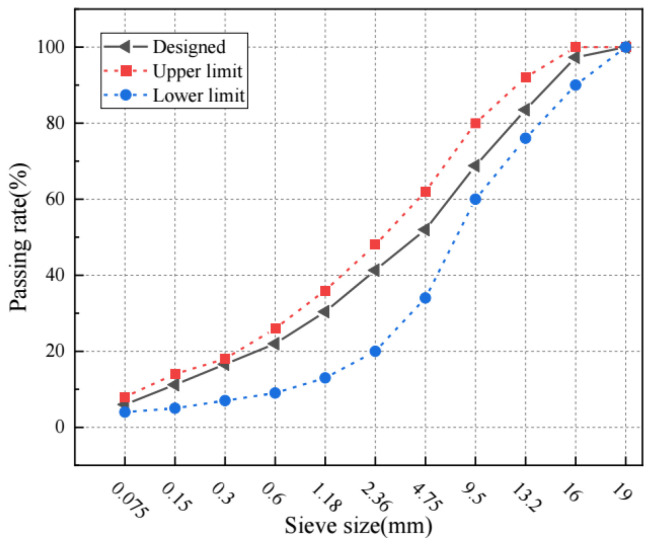
The aggregate gradation curve of AC-16 asphalt concrete.

In this study, PG 76-22 asphalt and basalt aggregates were used to prepare the asphalt concrete with an asphalt content of 4.3% by weight and target air void content of 6% by volume. The AC-16 aggregate gradation curve is shown in Figure 4.

Considering the feasibility of specimen preparation for different materials, the complex permittivity of asphalt concrete and asphalt binder was measured through the rectangular waveguide method [32], while the coaxial method [33] was used to measure the complex permittivity of basalt particles. A cylindrical asphalt concrete specimen with 100 mm in diameter and 150 mm in height was prepared by a Superpave gyratory compactor (SGC). Considering the impact of the heterogeneous structure of asphalt concrete on the results, three pieces of the cuboid specimen with a size of 71.8 mm × 33.8 mm × 10 mm, which were cut from the middle of the cylindrical specimen, were prepared to measure the complex permittivity of asphalt concrete. Consequently, the mean value of the complex permittivity for asphalt concrete was adopted in the following analysis. The asphalt binder was heated in the oven at 170 °C for 1 h until the asphalt binder was completely liquid. Then, the liquid asphalt binder was poured into a customized mold to prepare a 71.8 mm × 33.8 mm × 5 mm cuboid specimen. The asphalt concrete and asphalt binder specimens were placed into the waveguide fixture.

The coaxial method and waveguide method are currently widely used to gauge the permittivity of materials. Since the specimen preparation for the waveguide method is easier than for the coaxial method, the basalt particles applied in asphalt concrete could not meet the dimension of the waveguide fixture. Moreover, the properties of larger basalt particles obtained otherwhere could not be guaranteed to be consistent with the basalt used in asphalt concrete. Therefore, the coaxial method was adopted to prepare the basalt specimen for gauging the permittivity of basalt in this study. A coaxial ring with an inner diameter of 3.04 mm and an outer diameter of 7 mm was made and placed in a coaxial fixture after mixing basalt particles and paraffin in a ratio of 3:7. After the preparation of all the specimens was completed, the complex permittivity of these specimens needed to be determined by using a microwave network vector analyzer (E5071C, Keysight AGILENT, purchased by Shenzhen, China) with a measurement accuracy of 1 × 10^−4^ and measurement range from 300 kHz to 20 GHz. The initial frequency, terminal frequency and step increment were set to gauge the complex permittivity. It was noted that the permittivity measured by the coaxial method was not consistent with the permittivity of basalt but basalt–paraffin effective permittivity. Actually, the permittivity of basalt should be calculated based on the Bruggeman formula [34]. Then, the permittivity of basalt after conversion was adopted in the simulation.

**Figure 5:**
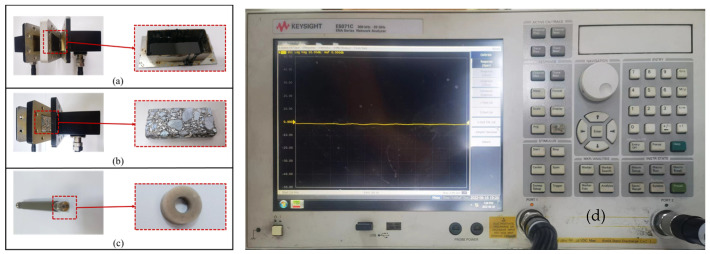
The tested specimen on the fixture for measuring complex permittivity and gauge equipment:(**a**) asphalt binder; (**b**) asphalt concrete; (**c**) basalt particles; (**d**) microwave network vector analyzer (E5071C).

The tested specimen on the fixture for measuring the complex permittivity and the gauge equipment are shown in Figure 5.

HD-32VNAWKN waveguide components were used in the laboratory due to the limitations of experimental instruments. As the dominant and foundational mode for the system’s operation [35], a TE_10_ waves mode with characteristics of a simple field structure, stability, a wide frequency band and low electromagnetic loss was adopted in this study. Therefore, the complex permittivity ranging from 2.6 to 3.9 GHz was gauged and simulated in this study.

### 4.2. The Analysis of Experimental Result

**Figure 6:**
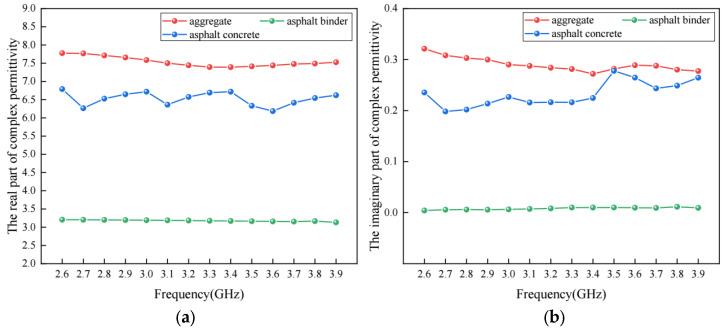
The complex permittivity of aggregate, asphalt binder and asphalt concrete: (**a**) real part; (**b**) imaginary part.

Based on the experiments above, the results for the real part and the imaginary part of the complex permittivity of basalt, asphalt binder and asphalt concrete are shown in Figure 6.

It is observed that the real part and the imaginary part of them are not constant. As shown in Figure 6a, the real part of the complex permittivity of basalt and asphalt concrete fluctuated in a range of 7.391–7.776 and 6.185–6.790, respectively, while, as shown in Figure 6b, a fluctuation could be found in the imaginary part of the complex permittivity of basalt and asphalt concrete with a range of 0.272–0.322 and 0.198–0.278, respectively. Compared to the aggregates and asphalt concrete, the real part and imaginary part of asphalt binder changed little with the increase in frequency. The variation can be considered a reasonable experimental error. Similar fluctuations to those above have also been observed in the previous literature [10,15] based on experiments. The reason for the phenomenon is related to the existence of air void, which leads to the change in the wave impedance of the material. Furthermore, it would have a significant effect on the impedance matching characteristics between the electromagnetic wave and the material. Multiple reflections and scatterings in the porous structure would occur for the electromagnetic wave. Moreover, the air void would induce interface polarization, resulting in a rise in the loss of the electromagnetic wave and a sudden change at a certain frequency point.

Though there are fluctuations with different degrees for the complex permittivity of basalt, asphalt binder and asphalt concrete, it is obvious that the real part and imaginary part of the complex permittivity of basalt are at their maximum, while for asphalt binder, they present as the minimum value.

## 5. Discussion

### 5.1. Model Validation

**Figure 7:**
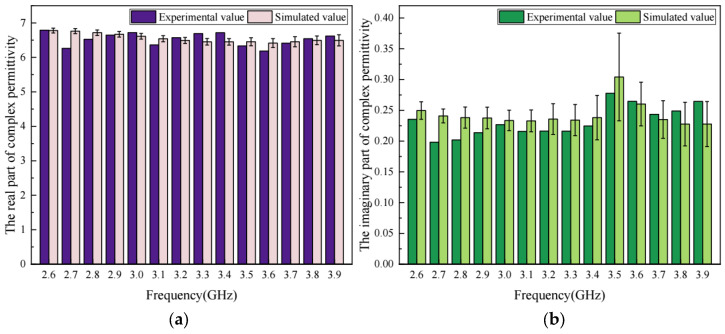
The comparison of experimental value and simulated value in complex permittivity for asphalt concrete: (**a**) real part; (**b**) imaginary part.

With the above 3-D meso-structure-based model, the complex permittivity of virtual asphalt concrete specimens was evaluated. The mean values in the real part and the imaginary part of the complex permittivity of asphalt concrete are presented in Figure 7, together with the values measured through experiments.

**Figure 8:**
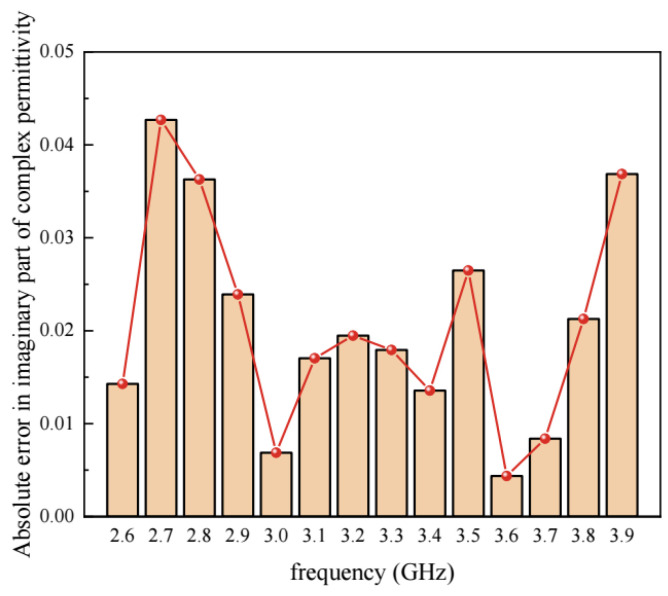
The absolute error in imaginary part of complex permittivity.

As shown in Figure 7, the maximum relative error between the simulated values and the experimental values in the real part of the complex permittivity of asphalt concrete is 7.9%, and the relative error at other frequencies is below 4%, while for the imaginary part of the complex permittivity, the maximum relative error is 19.6% and the average relative error is 9.01% at all frequencies. It is observed that the error in the imaginary part is larger than that in the real part. The main reason is that the value of the imaginary part is relatively small, which results in larger relative errors between the calculated and the measured imaginary part. Figure 8 shows the absolute error between the calculated and the measured values of the imaginary part. It proves that the maximum absolute error in the imaginary part is approximately 0.04. Therefore, it is believed that the accuracy of the dielectric model for asphalt concrete presented in this study is acceptable.

### 5.2. Effect of Porosity on Permittivity

**Figure 9:**
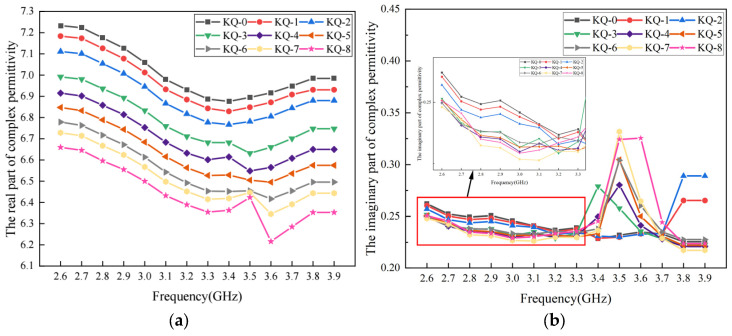
The complex permittivity of asphalt concrete with different porosities: (**a**) real part; (**b**) imaginary part.

In this section, the 3-D meso-structure-based model was used to analyze the effect of porosity on the complex permittivity of asphalt concrete. In total, nine sets of virtual asphalt concrete specimens with porosities ranging from 0 to 8% were modelled. The asphalt content and aggregate gradations in these virtual specimens were the same as those used in previous sections. These virtual specimens were marked as KQ-0, KQ-1, KQ-2, KQ-3, KQ-4, KQ-5, KQ-6, KQ-7 and KQ-8. Using the methods presented in this study, the complex permittivity of these specimens was calculated under different frequencies. The results of the real part and the imaginary part are shown in Figure 9.

As shown in Figure 9a, within the frequency range discussed in this study, the real part of the complex permittivity decreases with the increase in porosity. When the porosity is below 6%, the variation in the real part with frequency is relatively gentle, while sudden changes in the real part can be observed around the frequency of 3.5 GHz when the porosity is 7% and 8%. A similar trend is also observed for the imaginary part. As shown in Figure 9b, for the specimens with a relatively higher porosity, a sudden increase could be found in the imaginary part of the complex permittivity around some specific frequencies. The main reason for the above phenomenon is that the presence of the air void leads to the change in the wave impedance of the material, which would have a significant influence on the impedance matching characteristics between the electromagnetic wave and the material. Multiple reflections and scatterings in the porous structure would occur for the electromagnetic wave. Moreover, the air void would induce interface polarization, resulting in a rise in the loss of the electromagnetic wave and a sudden change at a certain frequency.

### 5.3. Effect of Void Size on Permittivity

**Figure 10:**
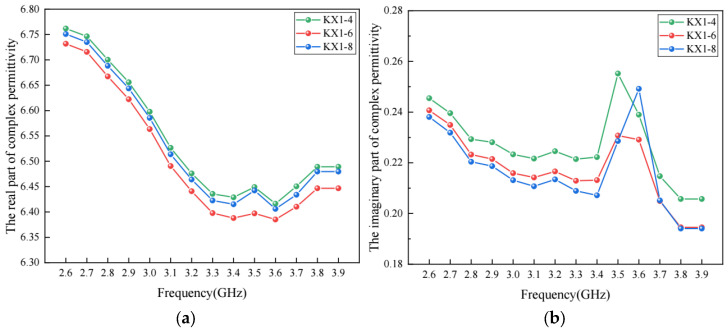
The complex permittivity for asphalt concrete with different void sizes: (**a**) real part; (**b**) imaginary part.

Besides porosity, the air void size may also have an impact on the complex permittivity of asphalt concrete. In this section, three virtual asphalt concrete specimens marked as KX1–4, KX1–6 and KX1–8 were developed with different air void sizes. The air void sizes in KX1–4, KX1–6 and KX1–8 ranged from 1 to 4 mm, 1 to 6 mm and 1 to 8 mm, respectively. The complex permittivity of these virtual specimens was calculated with the method presented in this study, and the results are shown in Figure 10. It is observed that air void size has an impact on the complex permittivity of asphalt concrete, but the effect of air void size is limited compared to porosity. In addition, Figure 10b shows that the air void size may have an effect on the frequency where the imaginary part has a sudden change. A larger air void size may lead to a larger frequency at which sudden change occurs.

### 5.4. Effect of Aggregate Gradation on Permittivity

**Figure 11:**
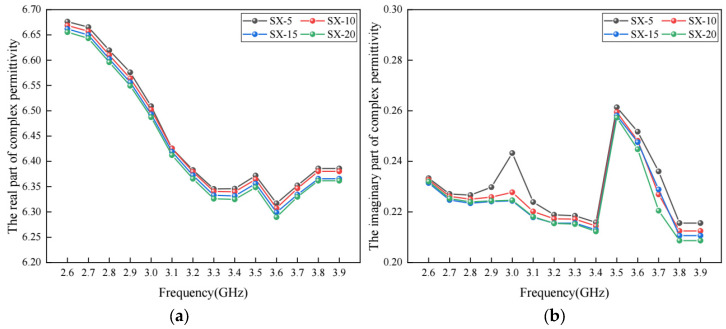
The complex permittivity with different aggregate gradation: (**a**) real part; (**b**) imaginary part.

Aggregate gradation has an important effect on various material properties of asphalt concrete. In this section, the 3-D meso-structure-based model was used to investigate whether aggregate gradation has a potential effect on the complex permittivity of asphalt concrete. To achieve this, part of the coarse aggregates in Table 1 was replaced with an equal volume of fine aggregate (with particle size of 2.36 mm). Four replacement rates, namely, 5%, 10%, 15% and 20%, were considered. With the changed aggregate gradation, four virtual specimens of asphalt concrete were developed, and were marked as SX-5, SX-10, SX-15 and SX-20, respectively. The complex permittivity of these virtual specimens was calculated, and the results are shown in Figure 11.

As shown in Figure 11a, the real part of the complex permittivity tends to be smaller when more coarse aggregates are replaced with fine aggregates. The main reason for this trend is that the absorption and loss capacity of coarse aggregates are stronger than those of fine aggregates, which is consistent with the findings reported in the previous literature [36]. At the same time, Figure 11b shows that the increase in fine aggregate content could also decrease the imaginary part of the complex permittivity.

### 5.5. Effect of Moisture Content on Permittivity

**Figure 12:**
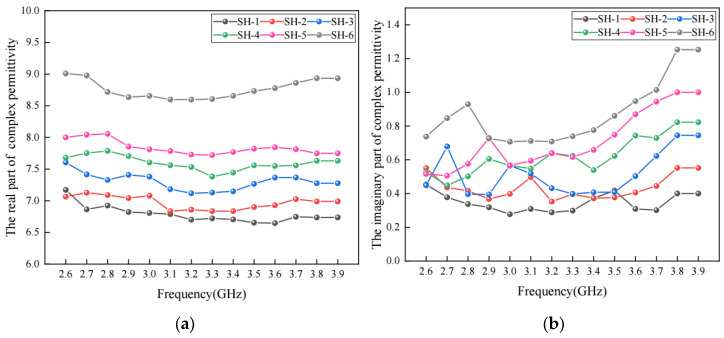
The complex permittivity of asphalt concrete with different moisture contents: (**a**) real part; (**b**) imaginary part.

When exposed to a humid environment, the moisture content inside the pavement structure increases. In order to investigate the effect of moisture on the complex permittivity of asphalt concrete, virtual asphalt concrete specimens with different moisture contents were developed. To achieve this, a reference virtual specimen with a porosity of 6% was generated first. By replacing some of the air voids with water, in total, six virtual specimens of asphalt concrete with moisture contents of 1%, 2%, 3%, 4%, 5% and 6% were obtained. These virtual specimens were marked as HS1, HS2, HS3, HS4, HS5 and HS6, respectively. The asphalt content and aggregate gradation in these specimens were kept the same. The complex permittivity of water was determined based on the previous literature [37]. The complex permittivity of the above specimens was calculated and the results are shown in Figure 12.

It can be found in Figure 12 that both the real part and the imaginary part of the complex permittivity increase with a higher moisture content, which is consistent with Reference [11]. This is mainly because the dielectric property of water is stronger than that of the air. Before all the air voids are filled with water, each 1% increase in moisture content would lead to about a 3~4% increase in the real part of the complex permittivity. When the moisture content increases from 5% to 6%, the increase in the real part of the complex permittivity is more obvious because all the air voids are filled with water. This is also consistent with Reference [12].

## 6. Modification of Dielectric Model

### 6.1. The Common Theoretical Models

(1) Lichtenecker–Rother (LR) model

The equation in the LR model is shown in Equation (14) [8] as follows:

(14) $$(\varepsilon_{m}{)}^{c}=\sum_{i=1}^{n}v_{i}(\varepsilon_{i}{)}^{c}$$

where *ε_m_*—The complex permittivity of the composed material;

*ε_i_*—The complex permittivity for the component *i*;

*ν_i_*—The volume fraction for the component *i*;

*C*—The influence coefficient for the geometric shape; 

*n*—The value of 4, component 1–4 represents aggregate, asphalt binder, void and moisture content.

The value of *c* is taken as 1, 1/2 and 1/3 for the general linear model (Brown model), the root-mean-square model (CRIM model) and the cube root model (Looyenga model), respectively. The equations are shown in Equations (15)–(17) as follows:

(15) $$\varepsilon_{m}=\sum_{i=1}^{n}v_{i}\varepsilon_{i}$$

(16) $$\sqrt{\varepsilon_{m}}=\sum_{i=1}^{n}v_{i}\sqrt{\varepsilon_{i}}$$

(17) $$\sqrt[{3}]{\varepsilon_{m}}=\sum_{i=1}^{n}v_{i}\sqrt[{3}]{\varepsilon_{i}}$$

(2) Rayleigh model

The expression [8] is shown in Equation (18) for multiphase substances as follows:

(18) $$\frac{\varepsilon_{m}-1}{\varepsilon_{m}+2}=\sum_{i=1}^{n}v_{i}\frac{\varepsilon_{i}-1}{\varepsilon_{i}+2}$$

### 6.2. The Applicability Analysis for Classic Models

**Figure 13:**
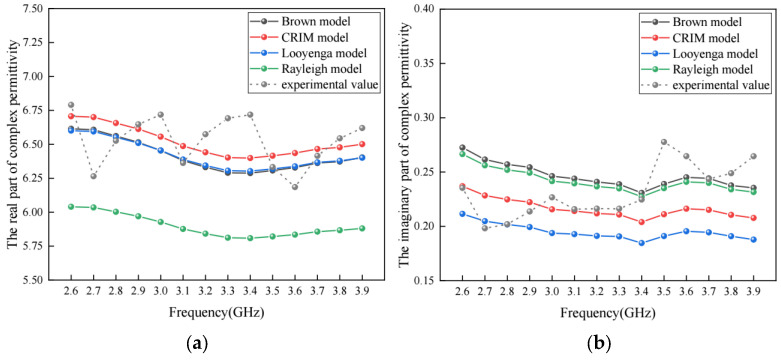
The comparison of complex permittivity between different classic models and experiment: (**a**) real part; (**b**) imaginary part.

The complex permittivity for four classic dielectric models (Brown model, CRIM model, Looyenga model and Rayleigh model) was calculated based on the complex permittivity of aggregates and asphalt binder obtained in the experiment and the volume fraction of each component with Equations (15)–(18). The real part and the imaginary part of the complex permittivity for the four models and the experiment are shown in Figure 13.

**Figure 14:**
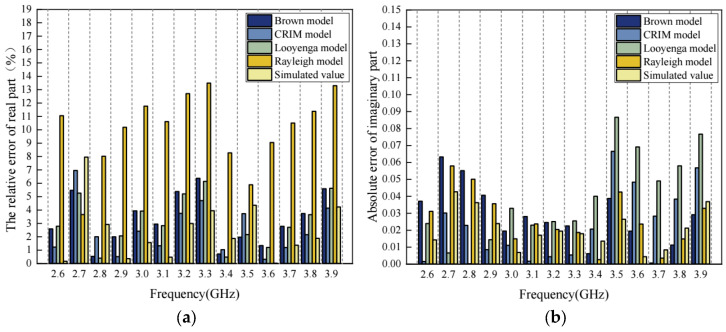
The errors of complex permittivity in different models: (**a**) the relative error for the real part of complex permittivity; (**b**) the absolute error for the imaginary part of complex permittivity.

For these classic models and the meso-structure model presented in this study, the relative error in the real part of complex permittivity is shown in Figure 14a, and the absolute error in the imaginary part of complex permittivity is shown in Figure 14b.

As shown in Figure 14a, the average relative errors in the real part of the complex permittivity are 3.24%, 2.53%, 3.17%, 9.99% and 2.34% for the Brown model, CRIM model, Looyenga model, Rayleigh model and the meso-structure model in this study. At the same time, Figure 14b shows that the maximum absolute errors in the imaginary part of the complex permittivity are 0.06326, 0.06657, 0.08665, 0.05795 and 0.04269 for the Brown model, CRIM model, Looyenga model, Rayleigh model, and the meso-structure model in this study. This indicates that the meso-structure model developed in this study has better accuracy compared to the classic models discussed in this section.

### 6.3. The Modified Equations Based on Meso-Scale Heterogeneous Model

In order to make the classical models more applicable to asphalt concrete, in this section, the coefficient in the LR model was modified based on the calculation results from the meso-structure model presented in this study. Twenty-four different conditions, shown in Table 2, were considered for conducting the calculation. In Table 2, the basic condition represents the asphalt concrete with an asphalt content of 4.5%, porosity of 6%, air void size between 4 and 8 mm and aggregate gradation as shown in Figure 1. Despite being the basic condition, the conditional variant shown in Table 2 highlights the differences between these conditions and the basic condition.

The complex permittivity of asphalt concrete shown in Table 2 was calculated with the meso-structure model presented in this study. The results were used to modify the classic models. The modified equations corresponding to the general linear model (Brown model), root-mean-square model (CRIM model) and cube root model (Looyenga model) are shown in Equations (19)–(21). The real part and the imaginary part of the complex permittivity were modified by the software IBM SPSS Statistics 26 for nonlinear fitting correction. The results are shown in Table 3.

(19) $$\varepsilon_{m}=\sum_{i=1}^{n}a_{i}v_{i}\varepsilon_{i}f$$

(20) $$\sqrt{\varepsilon_{m}}=\sum_{i=1}^{n}b_{i}v_{i}\sqrt{\varepsilon_{i}}f$$

(21) $$\sqrt[{3}]{\varepsilon_{m}}=\sum_{i=1}^{n}c_{i}v_{i}\sqrt[{3}]{\varepsilon_{i}}f$$

where *a_i_*, *b_i_*, *c_i_*, *d_i_*—The modified coefficient for each component in the Brown model, CRIM model and Looyenga model;

*n*—The value of 4, component 1–4 represents aggregate, asphalt binder, void and moisture content;
*f*—The tested frequency, GHz;
*ν_i_*—The volume fraction of each component;
*ε_i_*—The complex permittivity of each component.

**Table 3:** The modified coefficient of equations.

	Real Part of Complex Permittivity					Imaginary Part of Complex Permittivity				
Brown model	*a*_1_	*a*_2_	*a*_3_	*a*_4_	R^2^	*a*_1_	*a*_2_	*a*_3_	*a*_4_	R^2^
	0.338	−0.303	−1.538	0.073	0.922	0.285	0.552	0.171	0.171	0.980
CRIM model	*b*_1_	*b*_2_	*b*_3_	*b*_4_	R^2^	*b*_1_	*b*_2_	*b*_3_	*b*_4_	R^2^
	0.343	−0.766	−1.176	0.142	0.825	0.363	−0.350	0.457	0.454	0.974
Looyenga model	*c*_1_	*c*_2_	*c*_3_	*c*_4_	R^2^	*c*_1_	*c*_2_	*c*_3_	*c*_4_	R^2^
	0.342	0.939	1.309	0.176	0.690	0.383	−0.585	0.533	0.524	0.958

As shown in Table 3, among these modified classical LR dielectric models, the determination coefficients R^2^ for the real part and imaginary part fitted by the modified Brown model are the maximum values, which are 0.922 and 0.980, respectively.

## 7. Conclusions

In this study, an image-assisted approach was applied to generate 3-D geometries of aggregates, and then a multi-scale hierarchical modelling strategy was used to construct the virtual heterogeneous structure of asphalt concrete considering random distributions of components. The complex permittivity of the virtual specimens was numerically calculated in COMSOL. The accuracy of the meso-structure model was validated with experiments conducted in this study. Moreover, the effect of various factors on the complex permittivity was computationally analyzed, and then used to modify some classical models. The main conclusions include the following:

1. The maximum relative error between the calculated and measured real part of the complex permittivity was 7.9%, and the relative error at other frequencies was below 4%. The average relative error and maximum absolute error between the calculated and measured imaginary part of the complex permittivity were 9.01% and 0.04269, respectively. The accuracy of the meso-structure-based model is acceptable.
2. The real part of the complex permittivity of asphalt concrete decreased with the increase in porosity. Some sudden change in the imaginary part of the complex permittivity was observed within the frequency range from 2.6 GHz to 3.9 GHz. A larger air void size would lead to a larger frequency at which sudden change occurs.
3. The real part and the imaginary part of the complex permittivity tend to be smaller when more coarse aggregates are replaced with fine aggregates. This is mainly caused by the stronger absorption and loss capacity of coarse aggregates.
4. Both the real part and the imaginary part of the complex permittivity increase with higher moisture content due to the stronger dielectric property of water. Before all the air voids are filled with water, each 1% increase in moisture content leads to about a 3~4% increase in the real part of the complex permittivity. When all the air voids are filled with water, the above phenomenon is more significant.
5. The determination coefficients R^2^ for the real part and the imaginary part of the complex permittivity fitted by the modified Brown model were the maximum values, which were 0.922 and 0.980, respectively. The modified equations were more applicable for the calculation of the complex permittivity of asphalt concrete in the future.
6. Actually, temperature is a significant factor in the 3-D meso-scale modeling construction. However, the influence of temperature on the complex permittivity of asphalt concrete is not considered in this study. Moreover, the frequency range is relatively narrow. Therefore, these limitations will be taken into consideration in future work.

## Figures and Tables

**Table 1:** The dimensions of meso-scale hierarchical modelling.

Model Number	Composition	Model Dimension
Model 1	Asphalt binder + aggregates (smaller than 0.15 mm)	2 mm × 1.6 mm × 1.2 mm
Model 2	Asphalt binder + aggregates (smaller than 1.18 mm)	8 mm × 7 mm × 6 mm
Model 3	Asphalt binder + aggregates (smaller than 2.36 mm)	32 mm × 28 mm × 20 mm
Model 4	Asphalt binder + air void + aggregates (smaller than 2.36 mm)	70 mm × 60 mm × 10 mm
Model 5	Asphalt binder + air void + aggregates (in all sizes)	70 mm × 60 mm × 50 mm

**Table 2:** The simulated working conditions of complex permittivity for asphalt concrete.

Condition	Conditional Variant	Condition	Conditional Variant
1	Basic condition	2	Porosity of 0%
3	Porosity of 1%	4	Porosity of 2%
5	Porosity of 3%	6	Porosity of 4%
7	Porosity of 5%	8	Porosity of 6%
9	Porosity of 7%	10	Porosity of 8%
11	Porosity of 5%, moisture content of 1%	12	Porosity of 4%, moisture content of 2%
13	Porosity of 3%, moisture content of 3%	14	Porosity of 2%, moisture content of 4%
15	Porosity of 1%, moisture content of 5%	16	Porosity of 0%, moisture content of 6%
17	Heterogeneous distribution of 1–4 mm in void size	18	Heterogeneous distribution of 1–6 mm in void size
19	Heterogeneous distribution of 1–8 mm in void size	20	5% increasement in fine aggregate content
21	10% increasement in fine aggregate content	22	15% increasement in fine aggregate content
23	20% increasement in fine aggregate content	24	Same complex permittivity for each component

## Data Availability

Data are contained within the article.
